# Fast, low-memory detection and localization of large, polymorphic inversions from SNPs

**DOI:** 10.7717/peerj.12831

**Published:** 2022-01-20

**Authors:** Ronald J. Nowling, Fabian Fallas-Moya, Amir Sadovnik, Scott Emrich, Matthew Aleck, Daniel Leskiewicz, John G. Peters

**Affiliations:** 1 Electrical Engineering and Computer Science, Milwaukee School of Engineering, Milwaukee, Wisconsin, United States of America; 2 Electrical Engineering and Computer Science, University of Tennessee-Knoxville, Knoxville, Tennessee, United States

**Keywords:** Principal component analysis, Feature hashing, Chromosomal inversions, Single nucleotide polymorphisms

## Abstract

**Background:**

Large (>1 Mb), polymorphic inversions have substantial impacts on population structure and maintenance of genotypes. These large inversions can be detected from single nucleotide polymorphism (SNP) data using unsupervised learning techniques like PCA. Construction and analysis of a feature matrix from millions of SNPs requires large amount of memory and limits the sizes of data sets that can be analyzed.

**Methods:**

We propose using feature hashing construct a feature matrix from a VCF file of SNPs for reducing memory usage. The matrix is constructed in a streaming fashion such that the entire VCF file is never loaded into memory at one time.

**Results:**

When evaluated on *Anopheles* mosquito and *Drosophila* fly data sets, our approach reduced memory usage by 97% with minimal reductions in accuracy for inversion detection and localization tasks.

**Conclusion:**

With these changes, inversions in larger data sets can be analyzed easily and efficiently on common laptop and desktop computers. Our method is publicly available through our open-source inversion analysis software, Asaph.

## Introduction

Several methods and associated software packages for detecting and localizing large (>1 Mbp) polymorphic inversions from genome-wide single-nucleotide polymorphism (SNP) data are available (Sindi & Raphael, 2010; Ma & Amos, 2012; Zheng et al., 2012; Cáceres & González, 2015; Luu, Bazin & Blum, 2016; Love et al., 2019; Privé et al., 2020) and have been applied successfully to species such zebra finches (Knief et al., 2016), Atlantic cod (Kirubakaran et al., 2016), and sunflowers (Huang et al., 2020). Mutations that develop in a particular orientation are often private to that orientation. Recombination during meiosis is repressed in the inversion region between chromosomes with different inversion orientations (Noor et al., 2001; Rieseberg, 2001; Fuller et al., 2018), which prevents sharing of mutations. When variant sites are analyzed, they demonstrate high levels of correlation with each other and the inversion genotypes of the samples. Consequently, principal component analysis (PCA), which identifies groups of correlated variables and reduces each group to a single variable (component), is quite effective at identifying the presence of large inversions in a population. Since other processes such as population structure can also induce correlation, PCA can be combined with single-SNP association tests to localize the correlated SNPs and differentiating inversions from other sources of correlation (Nowling & Emrich, 2018; Nowling, Manke & Emrich, 2020).

It is not uncommon for variant data sets to have hundreds of thousands or even millions of SNPs per chromosome. For example, the *Drosophila* Genetics Reference Panel v2 (Huang et al., 2014) has ~750–950 k SNPs per chromosome arm, while 81 Burkina Faso *Anopheles gambiae* mosquito samples from the 1000 *Anopheles* Genome Project (Anopheles gambiae 1000 Genomes Consortium, 2017) have ~8.5–11.5 million SNPs per chromosome arm. Widely used machine learning libraries such as scikit-learn expect feature matrices to be encoded using 32-bit floating-point numbers. The amount of memory required to even load and construct feature matrices from these data sets can exceed what is available on current “high-end” desktops (*e.g*., 32 or 64 GB of RAM). For example, Tsuyuzaki et al. (2020) reviewed 21 implementations of 10 approximate PCA algorithms on single-cell RNA sequencing (scRNA-seq) data in terms of accuracy, run time, and memory usage; On the larger data sets, 13 PCA implementations required more than the available 128 GB of RAM and crashed.

While many common analyses such as inferring population structure (Patterson, Price & Reich, 2006; Reich, Price & Patterson, 2008), correcting for stratification in GWAS (Price et al., 2006), and detecting inversions (Ma & Amos, 2012; Nowling & Emrich, 2018) employ dimensionality reduction techniques such as PCA as a final step, they still require the construction of the full feature matrix in memory as a preliminary step. We are led to ask: why do we even need the full feature matrix in the first place if we are just going to perform PCA in the end? While efficient approximate PCA algorithms (*e.g*., Halko et al. (2011)) are commonly available in libraries like Scikit-Learn (Pedregosa et al., 2011), most methods are focused on reducing run time complexity, not memory usage. An out-of-core (online) PCA algorithm by Halko et al. (2011) and three online algorithms implemented in Tsuyuzaki et al. (2020) are most similar to our work in terms of their goals. The algorithm by Halko et al. (2011) requires the construction of ≥2 *O(mk)* matrices to find *k* components of a *n × m* matrix, which prevent any significant reduction in memory usage for wide data sets like those found in genomics. Tsuyuzaki et al. (2020) implemented three online PCA methods that were able to successfully process even the largest of the scRNA-seq they considered. Their online PCA methods, however, are only suited for streaming over samples (row first), while we need to be able to stream over variants (column-first) to detect inversions.

Diaz-Papkovich et al. (2019) demonstrated the feasibility of “stacking” multiple rounds of dimensionality reduction. They pre-processed their data with PCA, which is more computationally efficient, before performing a second round of dimensionality reduction with UMAP (McInnes, Healy & Melville, 2018), which is less scalable. With this in mind, we designed and implemented a two-step approach that avoids the construction of the full feature matrix in memory. First, feature hashing (Weinberger et al., 2009; Attenberg et al., 2009; Freksen, Kamma & Larsen, 2018) is used to directly construct a reduced feature matrix as variant data are read column-wise from a VCF file. Feature hashing is a naïve dimensionality reduction method related to random projection techniques (Freksen, Kamma & Larsen, 2018; Achlioptas, 2001; Li, Hastie & Church, 2006). Feature hashing requires less memory and compute power than PCA but is not able to reduce the data to as few dimensions. Therefore, we apply PCA to the much smaller matrix as a second round of dimensionality reduction to identify a handful of dimensions which capture the inversions of interest.

Here, we demonstrate that inversions can be detected and localized using a much smaller matrix. We achieve similar accuracies with as few as 10,000 dimensions, a significant reduction from the original hundreds of thousands to millions of dimensions. Most dimensionality reduction methods applied to matrices already loaded into memory, but this negates any potential memory savings. Instead, we use feature hashing (Weinberger et al., 2009; Attenberg et al., 2009; Freksen, Kamma & Larsen, 2018) to directly construct feature matrices in a reduced dimensionality space. When evaluated on previously characterized *A. gambiae* mosquito and *D. melanogaster* fly inversions, memory usage of our software Asaph (Nowling & Emrich, 2018; Nowling, Manke & Emrich, 2020) was reduced by up to 97% with no apparent reduction in accuracy.

## Materials and Methods

### Data sets

We used SNP data from the 2L and 2R chromosome arms of 81 *A. gambiae* and 198 *D. melanogaster* samples (see Table 1). These SNPs were originally released as part of the 1000 *Anopheles* Genome (Anopheles gambiae 1000 Genomes Consortium, 2017) and *Drosophila* Genetics Reference Panel v2 (Mackay et al., 2012; Huang et al., 2014) publications. We prepared the data (*e.g*., selected samples, kept only biallelic sites) by following steps described in Nowling, Manke & Emrich (2020) and as implemented in scripts available in the Asaph GitHub repository.

**Table 1:** Benchmark Results.

Species	Inversion	Samples	Number of SNPs	Inversion frequencies	Data source
*An. gambiae*	2La	81	8,296,600	90.7%	Anopheles gambiae 1000 Genomes Consortium, 2017
*An. gambiae*	2Rb	81	11,332,702	82.1%	Anopheles gambiae 1000 Genomes Consortium, 2017
*D. melanogaster*	*In(2L)t*	198	910,880	14.4%	Mackay et al., 2012; Huang et al., 2014
*D. melanogaster*	*In(2R)NS*	198	740,948	12.1%	Mackay et al., 2012; Huang et al., 2014

### Inversion analysis using SNP data

**Figure 1:**
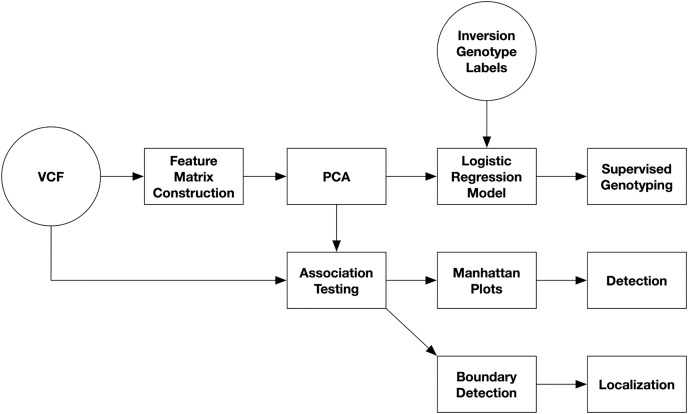
Diagram of inversion analysis workflows. In the first step of the analysis, SNPs are streamed from a VCF file and used to construct a feature matrix. In the second step, PCA is performed on the feature matrix. After this, there are three separate analyses that can be performed: supervised prediction of inversion genotypes using PC coordinates, detection of inversions from Manhattan plots visualizing PC-SNP association tests along the chromosome (arm), and localization of inversions by through boundary detection.

An overview of the pipeline for inversion analysis from single-nucleotide polymorphism (SNP) data is presented in Fig. 1. SNP data are first read from VCF files. The variant call format (VCF) is a tab-separated text file with headers for storing genomic variants for each sample in a population (Samtools, 2020). Each line stores the information for a single variant with its columns containing the values for each sample. Secondly, a feature matrix is constructed. Third, PCA is performed on the feature matrix. And lastly, inversions are detected and localized from Manhattan plots of PC-SNP associations.

### Feature matrix construction with categorical features

Assume that we have *n* samples and *v* positions with biallelic genotypes. Each position has a reference allele and an alternative allele, and at each position, each sample has one of three genotypes (homozygous reference, homozygous alternate, or heterozygous). We encode the variants as a feature matrix ***X*** with dimensions *n × 3v*. If sample *i* has the homozygous reference, homozygous alternate, or heterozygous genotype at position *k*, then we set matrix entry ***X***_*i,3k+1*_, ***X***_*i,3k+2*_, or ***X***_*i,3k+3*_, respectively, to 1. If the sample’s genotype is unknown, then all three entries are 0. The total required storage is *O(nv)*.

### Feature matrix construction with feature hashing

We create an *n × k* feature matrix ***X***, where *k* is a parameter provided by the user. The alleles for each variant position were encoded as strings in the form “chromosome_position_allele” (*e.g*., “2L_34534_T” or “2R_9897_A”). The associated column *p* for each allele was determined from the string *s* by *p = abs(hash(s)) % k* as described by Weinberger et al. (2009) (Note that we do not alternate the signs of the values). We increment the feature matrix entry ***X***_*i,p*_ by the number of copies sample *i* has of that allele. If the sample has a homozygous genotype, then the position will be incremented by 2. If the sample has a heterozygous genotype, then two positions are each incremented by 1. If the sample’s genotype is unknown, then no entries in the matrix are changed. Dimensionality reduction is achieved by setting *k* such that *k ≪ v*, the number of variant positions.

Our implementation uses the Scikit-Learn FeatureHasher transformer. The FeatureHasher API expects a list of all strings for each sample, while VCF files are transposed relative to the usual ordering of feature matrices; each line stores the alleles for all samples for a single variant. Our implementation proceeds as follows: A dense *n × k* feature matrix of all zeros is initialized. Lines (each containing one variant) of the VCF file are read and parsed in batches. A list of allele strings is created for each sample in the batch. The lists of strings are hashed and a sparse feature matrix of the hashed features for the subset of variants in the batch are returned. The sparse matrix is used to update the dense matrix and then discarded. This process is repeated until all variants are processed. The total required storage is *O(nk)*.

### Choosing the number of dimensions

Assume that we want to bound the ratio of distances in projected and full spaces between any two samples *u* and *v* by $\epsilon$:

$1 - \; \epsilon \; \le \displaystyle{{\parallel p\left( u \right) - p{{\left( v \right)}\parallel^2}} \over {\parallel u - {v\parallel^2}}} \le 1 + \epsilon$

The Johnson–Lindenstrauss (JL) lemma (Larsen & Nelson, 2017) relates the minimum number of dimensions *k* needed in a randomly generated subspace to bound the error by $\epsilon$ given the number of samples *n*:

(1) $$k \ge \displaystyle{{4{\rm log}\left( n \right)} \over {\displaystyle{{{\epsilon ^2}} \over 2} - \displaystyle{{{\epsilon ^3}} \over 3}}}$$

Assume that there are *l + m* SNPs. *l* SNPs are different between the inversion genotypes, while the genotypes of the *m* remaining SNPs are determined randomly with the caveat that there must be variation at each site. Assume that samples *u* and *v* both have one homozygous genotype and *w* has a heterozygous genotype of the inversion. The *l* SNPs will be the same in *u* and *v* but different between *w* and the other two:

$\parallel u - {v\parallel^2} \lt\parallel u - {w\parallel^2}\;$

We want to ensure that the bound $\epsilon$ on the error due to the projection is less than the ratio of samples with different inversion genotypes. We will need to pick a value for $\epsilon$ such that

$\epsilon < \displaystyle{{\parallel u - {w\parallel^2}} \over {\parallel u - {v\parallel^2}}} - 1$

We assume that the SNP genotypes are encoded as described above. In other words, one column is used per possible value of the variable and only one column in the group may have a value of 1 indicating that the variable has that value. For samples with unknown genotypes, all columns in the group will have values of 0. The maximum difference in distance between two samples contributed by a single variant position is 1. We assume that the *m* non-inversion SNPs are the same in the two samples *w* and *v* and any differences are among the *l* inversion SNPs. The total difference in distance due to the inversion is then:

$\epsilon < \displaystyle{{l + m} \over m} - 1$

(2) $$\epsilon < \displaystyle{l \over m}$$

Eq. (2) can be used to choose the $\epsilon$ parameter for Eq. (1) and estimate the minimum number of dimensions required to detect an inversion.

### Evaluation of inversion genotype separation with number of dimensions

The ability of inversion genotypes to be separated after PCA was evaluated using a supervised learning experiment (Nowling, Manke & Emrich, 2020). A sweep was performed over the dimensions parameter with the goal of determining whether the inversion genotypes were separable after PCA. For each number of dimensions, the following steps were taken: (1) a feature matrix of the given dimensionality was constructed, (2) the first two principal components were extracted using scikit-learn’s PCA class with the parameter whiten = True, (3) the samples were divided into training and testing sets using scikit-learn’s train_test_split function stratification enabled, (4) a logistic regression model was trained to predict the inversion genotypes from the first two PC coordinates using scikit-learn’s SGDClassifier class with the loss = “log” parameter, and (5) accuracy was calculated on the models’ predictions for the testing using scikit-learn’s accuracy_score function.

### Inversion detection

We compared Asaph, pcadapt (Luu, Bazin & Blum, 2016; Privé et al., 2020), and inveRsion (Cáceres et al., 2012; Cáceres & González, 2015; Caceres, 2021) on the task of inversion detection. SNPs were evaluated for association with the PC coordinates by statistical tests. The SNPs were read from the VCF file in a streaming fashion to avoid loading the full data set into memory. For each SNP, the genotypes of the samples were tested against the samples’ coordinates along a single principal component. We employed the one-way analysis of variation (ANOVA) test as implemented by Scipy (Virtanen et al., 2020). Samples’ PC coordinates were partitioned into groups by the samples’ genotypes. When a sample had a missing genotype for a given SNP, the sample was excluded from the test. The *−log*_*10*_ transformed *p*-values for each SNP were plotted along the chromosome to create a Manhattan plot. Inversions were detected by visual identification of a square wave pattern.

We used the following parameters for Asaph: Import and PCA using a reduced matrix was specified by the “--feature-type hashed --num-dimensions” flags to the asaph_pca program. 3,847 and 4,532 dimensions were used for the *A. gambiae* and *D. melanogaster* samples, respectively. With the full matrix, we used categorical features (“--feature-type categories”). PCA was performed with the default number of PCs (10) for Asaph. Only the first PC was used in association testing and plotting (“--components 1”).

For pcadapt, the VCF files were converted into the Plink binary bed (Purcell et al., 2007; Chang et al., 2015) format using Plink v1.9 (“–make-bed–allow-extra-chr”). Two principle components were specified for the analysis (“pcadapt (filename, K = 2)”). Lastly, SNP association scores were plotted (“plot (x, option = “scores”)”).

We used the following approach for the R package inveRsion: VCF files for the four SNP data sets were converted to the “raw” text format using Plink with “--recode A --allow-extra-chr”. A custom script (see Asaph GitHub repository) was used to convert the raw format to the expected file format for inveRsion. InveRsion was run on each of the four data sets with the following commands and parameters: setUpGenoDatFile (file = “inveRsion.txt”, sortMinor = TRUE, saveRes = FALSE), codeHaplo (gDat, saveRes = FALSE), scanInv (hapCode, window = 0.5, saveRes = FALSE), and listInv (scanRes, hapCode = hapCode, geno = TRUE, all = FALSE, thBic = 0). We encountered errors on the *Anopheles* data sets, but we were able to resolve the errors by specifying the regions of interest for the listInv function as 1 Mb to the left and right (19.5–43.5 and 18.0–28.0 Mbp, respectively) of the known 2La and 2Rb inversion boundaries from Corbett-Detig et al. (2019) and Lobo et al. (2010).

### Inversion localization

In Asaph v2, we introduced an algorithm for detecting inversion boundaries. The goal of the algorithm is to identify change points in between inversion and non-inversion regions. The SNP *p*-values from the PC–SNP association tests (see above) are used as input to the algorithm. Each SNP is categorized as significant or not using a Bonferroni-corrected significance threshold of 0.01/num_snps. The chromosome is divided into non-overlapping windows (default window size of 10 kb). The fraction of significant SNPs in each window is tested using a binomial test with the alternative hypothesis that the observed fraction of statistically significant SNPs is greater than expected. The expected probability of success (that a SNP is significant) is estimated as the fraction of statistically significant SNPs across the entire chromosome. In cases where a window has no SNPs or no significant SNPs, the *p*-value is estimated as 1.0. Windows are tested for significance using a Bonferroni-corrected significance threshold of 0.0001/num_windows. Lastly, the inversion ends are estimated from the centers of the left-most and right-most statistically significant windows.

We compare the predicted coordinates for the four inversions from Asaph v2 and inveRsion (Cáceres et al., 2012; Cáceres & González, 2015) to the known inversion boundaries coordinates from Corbett-Detig et al. (2019), Lobo et al. (2010), and Huang et al. (2014). We calculated the overlap between predicted and known coordinates (rounded to units of 0.1 Mb) using the Sørensen–Dice coefficient:

$DSC = \; \displaystyle{{2\left| {P \cap T} \right|} \over {\left| P \right| + \left| T \right|}}$

where P is the range of the predicted region in units of bp and T is the range of the known region in units of bp.

### Benchmarking

We benchmarked Asaph (with full and reduced matrices), pcadapt, and inveRsion on detection and localization to measure run times and memory usage. Note that the workflows for detection and localization are the same for Asaph so this workflow was only run once for both tasks. The separate stages of the Asaph, pcadapt, and inveRsion workflows were grouped into single tasks as shell and R scripts, respectively. We used the same parameters used in the validation analyses as described above. Run times and memory usage were recorded with the GNU time command (v1.7) using the “-v” flag. The following software versions were used in the benchmarks: Python 3.7.3, R 3.6.3, matplotlib 3.3.2, Numpy 1.19.2, scipy 1.5.2, seaborn 0.11.0, and sklearn 0.23.2. Conversion from VCF to Plink bed (pcadapt) and custom text file formats (inveRsion) were not included in the benchmarks.

### Software implementation

This software, which we have named Asaph, is available on GitHub (https://github.com/rnowling/asaph) under the open-source Apache Software License v2.0. Asaph is implemented in Python 3 and uses the Numpy (Harris et al., 2020), Scipy (Virtanen et al., 2020), and Scikit-Learn (Pedregosa et al., 2011) libraries. Asaph development is supported by a series of tests written against the command-line interface and run automatically upon commit by a continuous integration (CI) process. Documentation is provided in the form of tutorials available in the repository.

## Results

### Heuristic accurately estimates number of dimensions required

**Figure 2:**
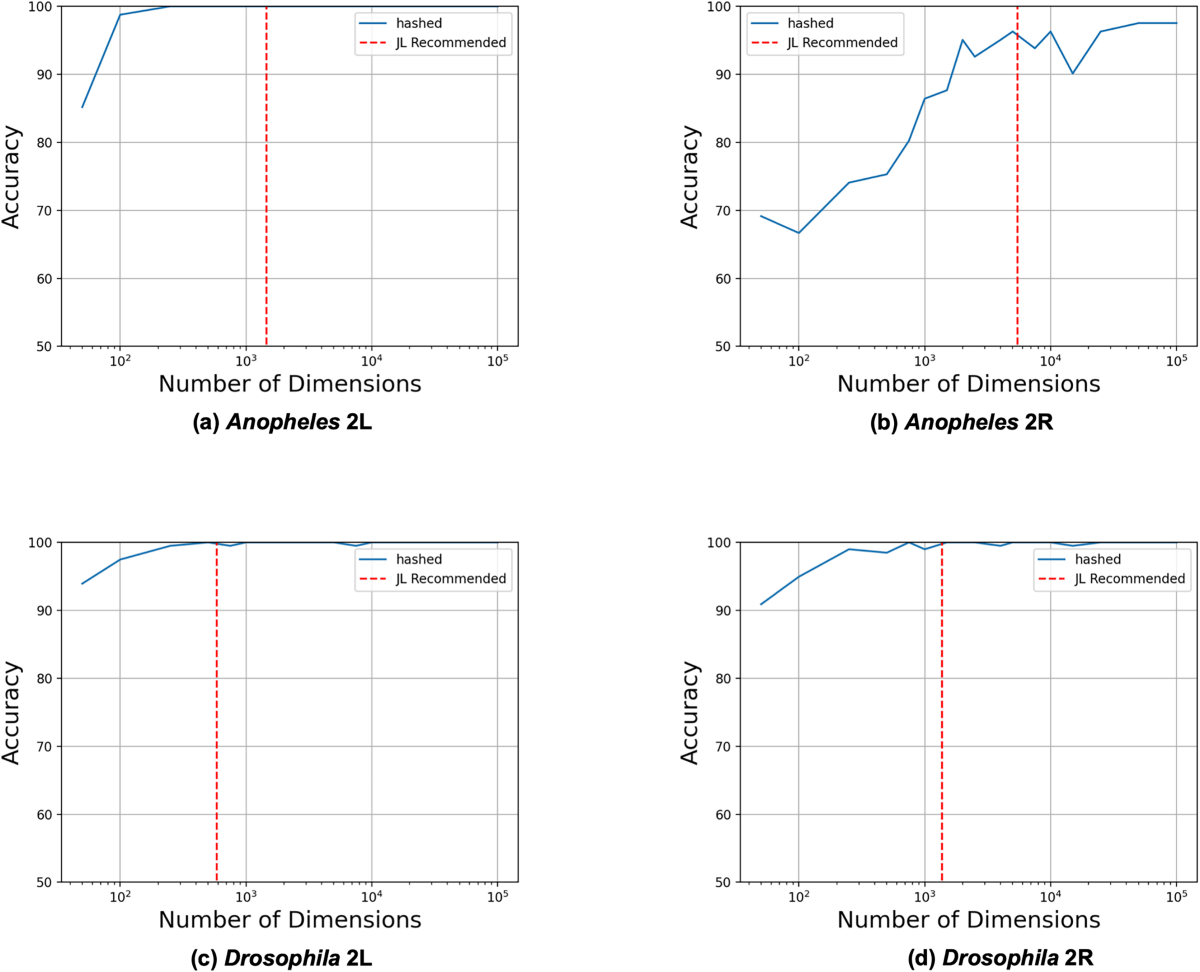
Heuristic accurately identifies upper bound on required dimensions. Feature matrices were constructed from SNPs from the four different data sets using feature hashing. PCA was performed on the hashed feature matrices, and coordinates along the first two principal components were used as inputs to logistic regression models trained to predict the inversion genotypes. The LR model was trained and evaluated with 10 to 10,000 feature hashing dimensions (blue solid lines). The maximum numbers of needed dimensions (red dashed lines) were estimated with the JL Lemma from the fraction of the chromosome spanned by the inversions according to their previously determined physical coordinates (see Methods section).

We began by evaluating how the number of reduced dimensions influenced the separability of the inversion genotypes with principal component analysis (PCA) (see Fig. 2). We used SNP data sets for four chromosome arms (2L and 2R of 81 *A. gambiae* (Anopheles gambiae 1000 Genomes Consortium, 2017) and 2L and 2R of 198 *D. melanogaster* (Huang et al., 2014)) each with a single large (>5 mb) polymorphic inversion (see Table 1). Using our derived heuristic (see Methods section), we estimated that 3,847 and 4,532 dimensions, respectively, would be required to analyze inversions in the *A. gambiae* and *D. melanogaster* samples. To evaluate this estimate, we constructed reduced feature matrices ranging from 10 to 10,000 dimensions, performed PCA, and trained logistic regression models to predict inversion genotypes from the first 2 PC coordinates. The model genotyped the *A. gambiae* 2La and *D. melanogaster In(2L)t* and *ln(2R)NS* inversions with 100% accuracy but only 94% accuracy for the *A. gambiae* 2Rb inversion. Significantly fewer dimensions were needed than estimated by our heuristic in all cases except for the *A. gambiae* 2Rb inversion, for which the estimate was similar to the observed number of required dimensions. We concluded that the heuristic model is a useful heuristic for estimating the required number of reduced dimensions.

### Accurate inversion detection with substantially less memory

**Figure 3:**
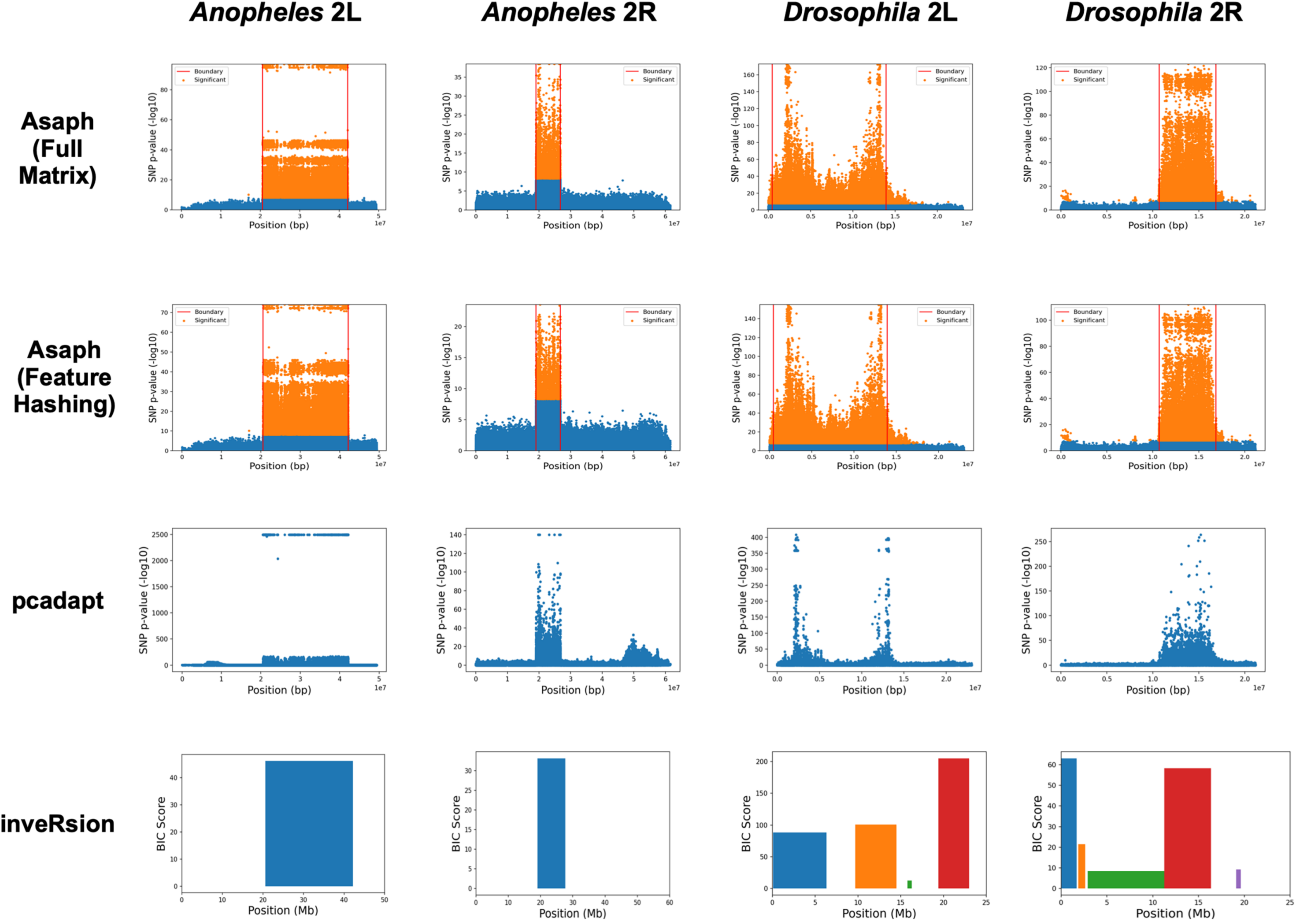
Comparison of predicted inversion regions. We evaluated Asaph (with full and reduced feature matrices), pcadapt, and inveRsion on the task of detecting inversions in four SNP data sets. Pcadapt and Asaph generate Manhattan plots showing −log_10_ *p*-values from association tests between PCs and SNPs, while inveRsion identifies regions and calculates a Bayesian Information Criteria (BIC) score. Higher indicates greater confidence in all cases. InveRsion reports ranges for each predicted inversion on a chromosome; the box for each predicted is colored so that it can be distinguished from the others.

We next evaluated unsupervised detection of the four inversions with and without dimensionality reduction (see Fig. 3). The four inversions were clearly visible in Manhattan plots generated for both methods from single-SNP association tests between samples’ PC coordinates and SNP genotypes (see Figs. 3A–3D, 3E–3H). There were no apparent differences in the Manhattan plots created with and without dimensionality reduction. We concluded that the online dimensionality reduction has no discernable negative impact on Asaph’s ability to detect inversions.

We next compared Asaph v2 to pcadapt (Luu, Bazin & Blum, 2016; Privé et al., 2020) and inveRsion (Cáceres et al., 2012; Cáceres & González, 2015; Caceres, 2021). Pcadapt identified all four inversions (see Figs. 3I–3L), however only the endpoints of the *In(2L)t* inversion were identified in Fig. 2K. We encountered an error in inveRsion during the “scanInv” step when attempting to scan the entire 2L and 2R chromosome arms for the *A. gambiae* data; we re-ran the step and defined a region of interest spanning 5 mb upstream and downstream of the known 2La and 2Rb inversion coordinates. InveRsion detected the 2La and 2Rb inversions as the only inversions (see Figs. 3M, 3N). InveRsion ran without error on the *D. melanogaster* 2L and 2R chromosome arms but detected multiple potential inversions on each arm (see Figs. 3O, 3P). For 2L, two (0.1–6.3 Mbp, 9.7–14.5 Mbp) of the four detected regions covered the left and right endpoints of the *In(2L)t* inversion. The two other regions (15.8–16.3 Mbp, 19.4–23.0 Mbp) were not associated with any of the inversions described by Huang et al. (2014). Five possible inversion regions were detected on 2R (see Fig. 3P). Huang et al. (2014) described seven other inversions (*In(2R)Y1*–*In(2R)Y7*) on 2R, each observed in only a single individual; yet none of the four other regions predicted by inveRsion were good matches. We concluded that Asaph and pcadapt were similarly effective in detecting inversions, while inveRsion was more sensitive at the cost of potential false positives.

### Inversion breakpoint localization with new boundary detection algorithm

In Asaph v1 and pcadapt, inversions are localized through visual inspection of the Manhattan plots. For Asaph v2, we designed a boundary detection algorithm (see Methods section) that provides precise predictions of the inversion breakpoint locations (see Table 2 and horizontal red lines in Figs. 3A–3H). We noted above that inveRsion detected the two endpoints of the *D. melanogaster In(2L)t* inversion as separate regions, so we merged these two regions to create a single predicted region (0.1–14.5 Mbp). For 2R, only a single predicted region (11.3–16.4 Mbp) overlapped with the known coordinates for *In(2R)NS* and was used.

**Table 2:** SNP data sets.

Chromosome	Inversion	Expected range (Mb)	Predicted range (Mb, Overlap)		
			Asaph (full)	Asaph (reduced)	inveRsion
*Anopheles* 2L	2La	20.5–42.2	20.5–42.2 (100.0%)	20.5–42.2 (100.0%)	20.6–46.2 (91.3%)
*Anopheles* 2R	2Rb	19.0–26.8	19.0–26.7 (99.3%)	19.0–26.7 (99.3%)	19.1–27.7 (93.9%)
*Drosophila* 2L	*In(2L)t*	2.2–13.2	0.5–14.3 (88.7%)	0.5–13.9 (90.2%)	0.1–14.5 (86.7%)
*Drosophila* 2R	*In(2R)NS*	11.3–16.2	10.7–16.5 (91.6%)	10.7–16.9 (88.3%)	11.3–16.4 (98.0%)
**Average** **Overlap**			94.9%	94.5%	92.5%

**Note:**

In this study, we used four SNP data sets drawn from two chromosome arms of two insects. Details of the data sets, inversions, and sources are detailed below.

We evaluated agreement by calculating overlap (using the Dice coefficient) between the predicted coordinates from Asaph v2 (with full and reduced feature matrices) and inveRsion and the physical coordinates given in Lobo et al. (2010), Huang et al. (2014), and Corbett-Detig et al. (2019) Asaph achieved a slightly higher average overlap across all four inversions with the full matrix (94.9% for the full matrix, 94.5% for the reduced matrix). The differences manifested in improved overlap for *D. melanogaster In(2R)NS* (91.6% *vs* 88.3%) but worse overlap for *D. melanogaster In(2L)t* (88.7% *vs* 90.2%); Asaph identified the same boundaries for *A. gambiae 2La* and 2Rb with both matrices. When compared with inveRsion, the difference in average overlap between the two methods was relatively small (94.5% for Asaph with the reduced matrix, 92.5% for inveRsion). Notably, inveRsion was more accurate for the *D. melanogaster In(2R)NS* inversion, while Asaph v2 was more accurate for the remaining three inversions (*A. gambiae 2La and 2Rb* and *D. melanogaster In(2L)t*).

### Online dimensionality reduction substantially reduces memory usage

We benchmarked the three tools (Asaph with full and reduced feature matrices, pcadapt, and inveRsion) across three tasks (detecting, localizing, and genotyping inversions) and recorded their run times and memory usage (see Table 3). In cases where a tool did not support a particular task, a benchmark was not performed. In particular, pcadapt does not support localization or genotyping, while inveRsion does not support genotyping. Note that the workflows for detection and localization with Asaph are the same, so a single benchmark was performed for both tasks.

**Table 3:** Estimated inversion breakpoints.

Data set	Method	Detection	Localization	Run time (s)	Memory usage (GB)
*An. gambiae* 2L	Asaph (full)	x	x	5 m 31 s	10.0 GB
	Asaph (reduced)	x	x	5 m 26 s	0.3 GB
	pcadapt	x		10 s	0.6 GB
	inveRsion	x	x	4 h 13 m	13.9 GB
*An. gambiae* 2R	Asaph (full)	x	x	7 m 0 s	13.5 GB
	Asaph (reduced)	x	x	7 m 36 s	0.3 GB
	pcadapt	x		14 s	0.8 GB
	inveRsion	x	x	11 h 1 m	19.7 GB
*D. melanogaster* 2L	Asaph (full)	x	x	3 m 56 s	11.6 GB
	Asaph (reduced)	x	x	3 m 36 s	0.3 GB
	pcadapt	x		10 s	0.5 GB
	inveRsion	x	x	3 h 1 m	9.0 GB
*D. melanogaster* 2R	Asaph (full)	x	x	3 m 14 s	9.5 GB
	Asaph (reduced)	x	x	3 m 10 s	0.3 GB
	pcadapt	x		9 s	0.4 GB
	inveRsion	x	x	2 h 33 m	8.6 GB

**Note:**

We compared the accuracy of inversion localization (boundary detection) by Asaph (with full and reduced matrices) with inveRsion. Overlaps between the estimated and predicted ranges were calculated using the Dice coefficient.

Pcadapt was the fastest (≤14 s) tool for detecting inversions across all four data sets, while Asaph (with both feature engineering methods) had the second lowest run time (≤7 m 36 s). Asaph with the reduced feature matrix had the lowest memory usage (≤0.3 GB), while pcadapt’s had the second lowest memory usage (≤0.8 GB). InveRsion’s run time (≤11 h 1 m) and the memory usage for inveRsion (≤19.7 GB) and Asaph with the full feature matrix (≤13.5 GB) were several orders of magnitude greater than pcadapt and Asaph with the reduced feature matrix. Note that the same run time and memory usage results apply to Asaph and inveRsion for localization since the detection and localization workflows are the same for each program.

Overall, we conclude that the required run time and memory usage of pcadapt and Asaph with the reduced feature matrix are suitable for use on a common desktop or laptop computer and the differences between the two would be largely inconsequential to most users. Asaph with the reduced feature matrix offers a substantial improvement in run time compared with Asaph with the full feature matrix. InveRsion is not a viable option for users with common desktop or laptop computers due to extremely long run times and high memory usage.

## Discussion

We evaluated feature hashing for reducing the dimensionality of feature matrices generated from SNPs and the impact on the detection of large, polymorphic inversions from variant data. We described a heuristic that applies the Johnson–Lindenstrauss (JL) lemma to determine the number of dimensions used for feature hashing based on the expected size of the inversion relative to the size of the chromosome. When evaluated on two *Anopheles* and two *Drosophila* inversions, the heuristic appeared to overestimate the number of required dimensions in most cases (Overestimation is preferred to underestimation which would prevent the model from detecting the inversions).

With these changes, Asaph’s memory usage was reduced from tens of gigabytes to hundreds of megabytes (up to 97%) for the four data sets considered here. Asaph and pcadapt were similar in memory usage and run time and both were significantly faster and more memory efficient than inveRsion. Asaph and pcadapt employ different but complementary approaches. Pcadapt has native support for Plink’s binary bed format (Chang et al., 2015; Purcell et al., 2007), which is able to encode each genotype with just 2 bits. In comparison, libraries like Scikit-Learn operate on matrices that use a 4-byte (or 32-bit) floating point number to encode each genotype. From the storage format alone, pcadapt reduces memory usage by a factor of 16. Internally, pcadapt provides functions for calculating matrix-vector multiplications so that it can perform linear algebra operations without unpacking the data. Our online dimensionality reduction approach could be combined with pcadapt’s native support for the efficient binary Plink bed format to obtain even further reductions in memory usage.

We also introduced an automated boundary detection algorithm. Inversion regions predicted by the algorithm were able to reproduce the experimentally-determined region with an average accuracy of 94.5%. The boundary detection algorithm is a step towards producing a fully automated pipeline for inversion detection and localization that could be used for high-throughput annotation of polymorphic inversions from variant data sets.

That said, our method was only tested on four data sets from two insect species, *A. gambiae* and *D. melanogaster*. The inversions chosen as test cases could be considered relatively easy. The data sets had large sample sizes (81 and 198 samples, respectively), the inversions had relatively high frequencies (at least 10%), and only one inversion was present on each chromosome. Going forward, it would be useful to test more challenging cases such as multiple, overlapping inversions such as the 2Rbc inversion system in *A. gambiae* and the 3R chromosomal arm of *D. melanogaster* and smaller, lower-frequency, and more recently arisen inversions. We will also want to test alternatives to dimensionality reduction such as SNP thinning (*e.g*., selecting 1 SNP per 100 Kbp region) and the impact of data sets with large numbers of SNPs with unknown genotypes.

## Conclusions

In summary, Asaph can now analyze inversions using large variant data sets on a commodity desktop or laptop computer. By achieving these performance improvements with minimal changes in accuracy, we believe that Asaph will be of wider interest and see greater usage. Further, the online dimensionality reduction approach we outlined can be used to scale other variant data workflows (*e.g*., population inference) and has significant utility beyond just the Asaph software package.

## Additional Information and Declarations

### Data Availability

The following information was supplied regarding data availability: The source code is available at GitHub: https://github.com/nowling-lab/asaph and archived at Zenodo: RJ Nowling. (2021). rnowling/asaph: Asaph v2.0 beta (v2.0-beta). Zenodo. https://doi.org/10.5281/zenodo.4521676.
